# The Cytoplasmic Domain of MHC Class I Molecules as a Molecular Switch: A Perspective from Short Linear Motifs and Intrinsically Disordered Regions

**DOI:** 10.3390/biom16071067

**Published:** 2026-07-22

**Authors:** Fernando A. Arosa, Elsa M. Cardoso

**Affiliations:** 1 RISE-Health-UBI, University of Beira Interior, 6200-506 Covilhã, Portugal; 2 FCS-UBI, Faculty of Health Sciences, University of Beira Interior, 6200-506 Covilhã, Portugal; 3 BRIDGES, ESS-IPG, School of Health Sciences, Polytechnic of Guarda, 6300-035 Guarda, Portugal

**Keywords:** phosphorylation, flexibility, conformational changes, reverse signaling, inside-out signaling, MetaPredict, flDPnn, neurodegeneration

## Abstract

Classical Major Histocompatibility Complex Class I (MHC-I) molecules are traditionally viewed as stable peptide-presenting structures expressed on the surface of all nucleated cells. Their expression by professional antigen-presenting dendritic cells (DCs) enables CD8+ T-cell activation, differentiation, and immune surveillance. However, accumulating evidence indicates that cell-surface MHC-I molecules exist in three major conformational states: (1) β2m-associated, peptide-loaded conformers that originate in the endoplasmic reticulum and pass through the Golgi apparatus after binding proteasome-generated cytosolic peptides (hereafter referred to as closed conformers); (2) β2m-free, peptide-empty conformers that arise following β2m dissociation from closed conformers either at the plasma membrane or after internalization and recycling (hereafter referred to as open conformers); and (3) β2m-associated, peptide-empty conformers that represent an intermediate state between closed and open conformers. Here, we propose a conceptual framework, supported by computational predictors of intrinsically disordered regions, in which transitions between closed and open MHC-I conformers are coupled to intracellular regulatory processes, including post-translational modifications of conserved motifs, intracellular trafficking, and signaling. Although direct experimental evidence linking these processes remains limited, we integrate independent observations into a working model that may guide future investigations into MHC-I-mediated cell–cell communication in both immune and non-immune contexts, in health and disease. For clarity, in this article we define “open conformers” as structurally competent, β2m-free, and peptide-deficient MHC-I molecules.

## 1. Introduction

Over the past decades, MHC-I molecules have been primarily studied in the context of antigen presentation and immune surveillance, leading to a well-established structural and functional framework centered on peptide loading and CD8+ T-cell recognition. Within this paradigm, a growing body of experimental evidence from the 1990s and 2000s demonstrated that non-canonical conformational states of MHC-I molecules, including β2m-free and peptide-empty forms, can be detected on the surface of human cells under both physiological and stress-related conditions.

Notably, these conformational states have been linked to post-translational modifications (PTMs) within the cytoplasmic tail. These findings raise the possibility that conformational plasticity of both extracellular and intracellular domains contributes to previously unappreciated biological roles of MHC-I molecules. Rather than providing a comprehensive review of the literature, this Perspective focuses on a selected set of recent observations to develop a conceptual framework in which extracellular and intracellular MHC-I conformational dynamics are closely linked to regulatory processes, including PTMs such as tyrosine phosphorylation.

We propose that the physiological equilibrium between closed and open MHC-I conformers is modulated by the activation and metabolic state of the cell and that this dynamic balance may contribute to the integration of intracellular signaling, protein–protein interactions, and cell–cell communication. Although this model remains to be fully validated experimentally, it provides a conceptual basis for exploring the non-canonical functions of MHC-I molecules.

## 2. Extracellular Versus Cytoplasmic Domains of MHC-I Molecules

Classical MHC-I molecules (HLA-A, -B, and -C in humans) are highly polymorphic cell-surface glycoproteins that mediate presentation of peptides to CD8+ T cells. Structurally, MHC-I molecules consist of an ~45 kDa transmembrane α heavy chain polypeptide (hereafter αHC), non-covalently associated with an ~12 kDa β2-microglobulin light-chain polypeptide (hereafter β2m), and a small peptide of 8–15 amino acids. This MHC-I structure forms a trimeric complex that represents the canonical closed conformation [1]. The αHC has an extracellular region organized into three domains (α1, α2, α3), a transmembrane hydrophobic domain that holds the MHC-I molecule on the plasma membrane, and a short cytoplasmic tail. The α1 and α2 domains form the peptide-binding groove, whereas the α3 domain mediates interactions with the CD8 co-receptor, stabilizing T cell receptor (TCR) engagement [1].

**Figure 1:**
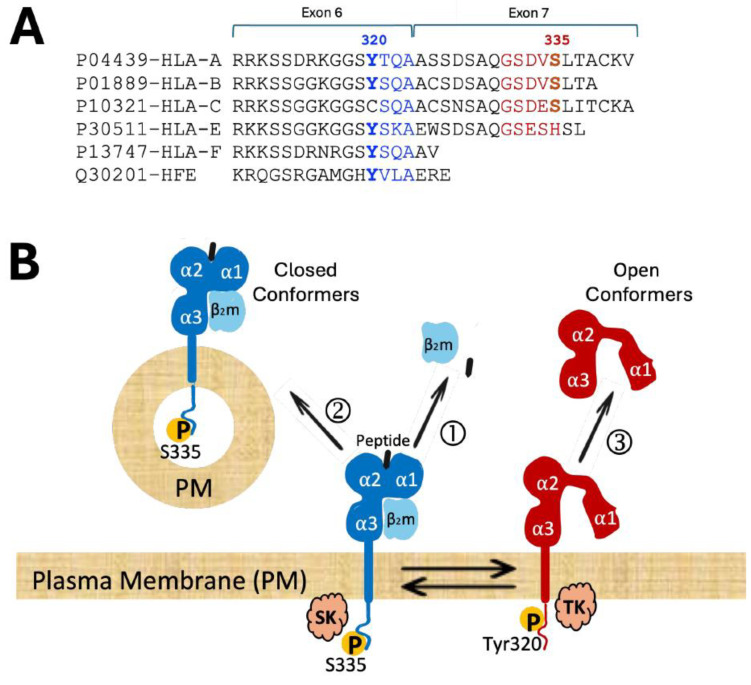
Proposed phosphorylation-dependent molecular switch regulating MHC-I conformational states. (**A**) Consensus amino acid sequences of the cytoplasmic domains of classical (HLA-A, HLA-B and HLA-C) and non-classical (HLA-E, HLA-F, HFE) molecules. Conserved SLiMs containing Y320 (blue) and Ser335 (red) are indicated. Exons 6 and 7 encoding these SLiMs are shown. (**B**) Schematic representation of the proposed relationship between post-translational modifications within the cytoplasmic domain and the extracellular conformational states of MHC-I molecules. Closed conformers consist of peptide-loaded, β2m-associated MHC-I molecules, whereas open conformers correspond to β2m-free and peptide-deficient heavy chains. Previous studies have shown that phosphorylation of Ser335 is associated with closed conformers, whereas phosphorylation of Tyr320 is associated with open conformers. Based on these observations, we propose that phosphorylation of conserved residues embedded within short linear motifs (SLiMs), shown in Figure 1A, functions as a molecular switch regulating the equilibrium between closed and open conformers. The scheme also shows the three possible fates of the plasma membrane MHC-I molecules: ① Release of β2m and the peptide from the closed conformers, which shifts the physiological equilibrium to the right, thus generating open MHC-I conformers. This shift occurs in activated and stressed cells and is closely linked to the phosphorylation of Tyr320 by a tyrosine kinase (TK), such as Lck in T cells, or another Src kinase in other cells; ② After endocytosis and intracellular recycling, vesicles/exosomes containing closed MHC-I conformers can be released into the extracellular space. Closed conformers are constitutively phosphorylated at Ser335 by a serine kinase (SK), such as PKA or PKC; ③ Release of soluble truncated forms of open MHC-I conformers, most likely after cleavage by a metalloprotease.

Although this closed conformation has long been considered the functional unit of MHC-I molecules and the focus of numerous studies, substantial evidence indicates that the extracellular α1 domain is endowed with conformational flexibility, which allows numerous conformational states to exist [2]. Thus, under physiological and stressed conditions, dissociation of β2m and/or peptide from the trimeric structure can give rise to cell- surface β2m- and peptide-free αHC forms, commonly referred to as open conformers [3] (see Figure 1). However, the field remains limited by the difficulty in clearly distinguishing between structurally defined naturally occurring open MHC-I species, which may lead to divergent interpretations of experimental data. Nevertheless, a recent study has elegantly demonstrated what was previously suspected, i.e., open conformers arise following β2m dissociation from trimeric forms located at the plasma membrane or after recycling from intracellular compartments [4]. The conformational plasticity of the extracellular domain of the αHC of human MHC-I molecules may be partly explained by its structural organization. In particular, the α1 domain, which forms part of the peptide-binding platform and lacks the stabilizing intradomain disulfide bond present in the α2 and α3 domains, may possess increased structural flexibility following β2m dissociation [5]. In addition, conserved cysteine residues within the α1, α2, and α3 domains may participate in intermolecular disulfide bond formation, potentially enabling the formation of homodimers or higher-order structures among open conformers [3,6]. It can be proposed that the transitions between closed and open conformers may affect not only peptide-binding capacity but also the spectrum of molecular interactions.

In contrast to the extracellular domain, relatively little research has addressed the conformational properties of the cytoplasmic domain of human MHC-I molecules. The cytoplasmic tail consists of a short polypeptide of ~30 amino acids containing short linear motifs (SLiMs), notably YXXA and GSDVS, which harbor the conserved phosphorylatable residues Tyr320 and Ser335 encoded by exons 6 and 7, respectively (Figure 1A). Interestingly, both SLiMs are thought to participate in the internalization of MHC-I molecules and other cell-surface receptors. The YXXA motif is highly reminiscent of tyrosine-based YXXØ endocytic motifs, in which X represents any amino acid and Ø denotes an amino acid with a bulky hydrophobic side chain, that are found in numerous cell-surface proteins and bind adaptor proteins [7]. However, the YXXA sequence represents a degenerated YXXØ motif due to the presence of a non-bulky alanine at the +3 position. Consequently, its binding affinity to clathrin adaptor proteins, such as the μ2 subunit of the adaptor protein complex 2 (AP2), is markedly reduced [8]. Interestingly, the two conserved SLiMs (i.e., TXXA and GSDVS) have been shown to serve as ligands for PDZ domains, named after the first three proteins identified to contain this domain: PSD-95, Dlg1, and ZO-1. PDZ domains bind conserved SLiMs located at the C-termini of target proteins, including MHC-I molecules, and are widely distributed in humans, where they form the backbone of scaffolds that organize cell–cell contacts, and regulate protein–protein interactions, protein localization, and binding specificity [9,10]. The presence of PDZ-binding motifs further suggests that the MHC-I cytoplasmic tail may function as a scaffold for signaling and trafficking complexes. Although several allelic variations exist in the cytoplasmic domain, particularly of HLA-B, its role as an independent disease variable remains poorly characterized and stands as an open question.

## 3. Effects of Phosphorylation and Dephosphorylation in the MHC-I Cytoplasmic Domain

A key conceptual advance in our understanding of the biology of cell-surface MHC-I molecules has been the recognition that closed and open MHC-I conformers segregate at the plasma membrane and can be internalized through clathrin-mediated endocytosis (CME) and clathrin-independent endocytosis (CIE), with marked variability across cell types. Although earlier studies suggested that MHC-I molecules followed the CME pathway, subsequent studies provided evidence that closed and open MHC-I conformers undergo distinct endocytic routes [11]. Closed MHC-I conformers exhibit a low rate of internalization, undergo CME, and localize to juxtanuclear recycling tubular endosomes together with the transferrin receptor, a well-characterized receptor that undergoes CME. In contrast, open MHC-I conformers exhibit a higher rate of internalization, undergo ARF6-dependent CIE, localize to vesicles outside the juxtanuclear tubular endosomes, and can reach late endosomal compartments [11,12,13,14,15]. In both endocytic pathways, MHC-I molecules can be sorted to late endosomes and multivesicular bodies, where they may undergo further degradation or processing, or contribute to secreted pools of vesicle-associated MHC-I molecules [13,16]. Nevertheless, although the Tyr320-containing YXXΦ motif provides a mechanistic basis for clathrin-mediated endocytosis, the molecular determinants responsible for the preferential routing of open MHC-I conformers through clathrin-independent endocytosis remain incompletely understood.

Regarding the SLiMs present in the cytoplasmic domain of MHC-I molecules, it is necessary to revisit old studies conducted during the 1980s and 1990s. Although several of these studies predate recent advances in the field, they provided original observations that, in part, motivated the present conceptual framework, namely the identification of cytoplasmic determinants associated with the closed and open MHC-I conformers and the importance of the cytoplasmic tail for constitutive endocytosis [17,18,19,20,21,22,23]. Some of these seminal studies demonstrated that the phosphorylation status of the Ser335 within the GSDVS motif was closely linked to the closed conformational state of the extracellular polymorphic region of human and mouse MHC-I molecules. In marked contrast, Tyr320 within the YXXA motif was later found to be phosphorylated in cell surface open MHC-I molecules expressed by activated T cells [24]. In this context, it can be noted that Tyr321, the murine equivalent of human Tyr320, was also found to be phosphorylated in lipopolysaccharide-activated macrophages [25].

Although Tyr320 within the YXXA sequence represents a degenerate YXXØ motif, recent studies using human and mouse antigen-presenting cells expressing either wild-type Y320 or the phosphomimetic mutant Y320E demonstrated that the Y320E substitution can drive CD8+ T cells toward a more potent and sustained effector phenotype after trafficking through endolysosomal compartments, where MHC-I molecules bind peptides derived from endocytosed exogenous antigens [26]. According to the authors, it is possible that the Y320E phosphomimetic substitution only weakly mimics the effects of Tyr320 phosphorylation, which may indicate that phosphorylated Tyr320 exerts an even stronger effect on CD8+ T-cell priming than that observed with the phosphomimetic mutant. However, the physiological implications of this observation remain to be demonstrated. In this regard, it is important to draw attention to earlier mutational studies in which thymoma cells transfected with mutant HLA-B27 molecules carrying a Tyr320Phe substitution (Y320F) exhibited impaired endocytosis, accumulation of HLA-B27 molecules at the cell-surface, and increased secretion of cleaved forms (~32 kDa), most likely originating from open HLA-B27 conformers [27].

According to current knowledge, these findings are noteworthy for three reasons. First, they suggest that preventing Tyr320 phosphorylation retains MHC-I molecules at the plasma membrane, thereby impeding or delaying their internalization. Second, phosphorylation-dependent alterations in cytoplasmic tail dynamics may influence the overall conformational landscape of MHC-I molecules and, consequently, indirectly affect the stability of extracellular MHC-I conformers. Third, these events, when considered together, may facilitate the establishment of cis interactions between MHC-I molecules themselves, leading to the formation of homodimers, or between MHC-I molecules and immune or non-immune receptors, leading to the formation of heterodimers [3,6]. Nevertheless, the implications of phosphorylation of the conserved tyrosine- and serine-containing PDZ-binding motifs for the conformational flexibility of the cytoplasmic tail of classical human HLA-I molecules remain largely understudied (see below). An important unresolved issue concerns the directionality of the relationship between Tyr320 phosphorylation and MHC-I conformational transitions. Current evidence does not distinguish whether Tyr320 phosphorylation promotes cytoplasmic domain conformational changes or whether extracellular domain opening exposes the cytoplasmic tail to phosphorylation. These possibilities are not mutually exclusive and may reflect a dynamic feedback mechanism operating at the plasma membrane. The possibility that a post-translational modification, such as Tyr320 phosphorylation, may trigger long-range allosteric changes that loosen the extracellular peptide-binding groove and thereby increase the propensity of MHC-I molecules to adopt open conformations has never been explored. If proven, this phenomenon would constitute a form of inside-out MHC-I signaling.

An important question arising from these observations is whether the conserved SLiMs containing Tyr320 and Ser335 function as phosphorylation-dependent molecular switches that regulate transitions between closed and open MHC-I conformations and thereby determine their cellular fate (Figure 1B). Phosphorylation is the most common post-translational modification in eukaryotes and occurs more frequently in intrinsically disordered proteins (IDPs), particularly within intrinsically disordered regions (IDRs) [28]. IDPs lack a fixed three-dimensional structure yet remain biologically functional. Their existence was first suggested by missing electron-density data in crystal structures, which were initially dismissed as experimental artifacts. The absence of electron density in crystal structures, which generally indicates that a protein region is highly flexible and therefore cannot be resolved in a fixed conformation, suggests that portions of the MHC-I cytoplasmic domain may be intrinsically disordered. IDPs came to be recognized as a distinct category of proteins in the late 1990s and early 2000s, when the classical “sequence → structure → function” paradigm, which assumes that a stable three-dimensional fold is a prerequisite for biological function, was shown to be incomplete. Indeed, approximately 30–40% of the human proteome consists of proteins or protein regions that lack stable tertiary structure under physiological conditions and exhibit a high degree of conformational flexibility while remaining biologically active [29]. In this context, two properties of IDRs are particularly relevant to the present Perspective and closely linked to the physiological equilibrium between closed and open MHC-I conformers: (1) IDRs exist as ensembles of interconverting conformations rather than adopting a single stable structure, thereby enabling them to adapt their shapes to bind different partners; and (2) phosphorylation frequently occurs in IDPs and IDRs and is commonly associated with conformational changes and fine-tuning of cellular signaling [30,31]. According to the DisProt database, approximately 1300 human proteins have been experimentally demonstrated to contain IDRs using low-throughput biophysical techniques such as nuclear magnetic resonance (NMR), circular dichroism, and X-ray crystallography. Surprisingly, to our knowledge, no evidence indicates that classical MHC class I molecules are listed as IDPs or contain annotated IDRs in the curated DisProt database, with the exception of the non-classical MHC-I molecule MICA (MHC class I polypeptide-related chain A). It should be noted that DisProt is a curated repository of proteins for which intrinsic disorder has been experimentally confirmed using low-throughput biophysical techniques. Therefore, the absence of classical MHC-I molecules from this database reflects a lack of systematic experimental investigation rather than confirmed evidence of a stable, ordered structure.

Although no direct evidence currently demonstrates long-range coupling between Tyr320 phosphorylation and extracellular conformational transitions, analogous phosphorylation-dependent allosteric mechanisms have been described in several proteins, supporting the plausibility of such a mechanism in MHC-I molecules. Thus, phosphorylation of tyrosine, serine and threonine residues has been associated with conformational changes in the IDRs of proteins, with important implications for downstream protein–protein interactions and signaling [28,29,30,31]. When extrapolated to MHC-I molecules, these observations support a model in which MHC-I molecules can be viewed as conformationally dynamic entities whose structural states may influence both extracellular and intracellular regulatory mechanisms. Nevertheless, direct mechanistic links between cytoplasmic tail phosphorylation and extracellular conformational transitions remain unclear. In this framework, the conserved SLiMs within the cytoplasmic domain of MHC-I molecules are not merely biochemical markers of closed and open conformers but active participants in the conformational switching of cell-surface MHC-I molecules (Figure 1B). Collectively, the observations summarized in Figure 1 support the concept that cell-surface MHC-I molecules exist as dynamic conformational states with distinct intracellular fates. Closed and open conformers differ in their endocytic pathways, trafficking behavior, and signaling potential [11,12,13,14,15,24,27], suggesting that conformational transitions are regulated processes rather than passive consequences of molecular instability. These findings provide the conceptual basis for proposing that MHC-I molecules function as a conformationally dynamic signaling system.

## 4. MHC-I Molecules as Intrinsically Disordered Proteins

As mentioned above, intrinsically disordered proteins lack a stable tertiary structure under physiological conditions and exhibit a high degree of conformational flexibility while remaining biologically active. Given the lack of published data regarding the presence of disordered regions in classical MHC-I molecules, we used the computational tools MetaPredict and flDPnn to predict IDRs in classical HLA-I molecules (HLA-A, HLA-B, and HLA-C). MetaPredict is a deep-learning-based bioinformatics tool designed to rapidly predict intrinsic disorder and structural confidence in proteins. It uses bidirectional recurrent neural networks trained on consensus disorder scores and predicts both per-residue intrinsic disorder and AlphaFold2 structural confidence (pLDDT) scores [32]. flDPnn is a deep neural network-based predictor specifically designed to identify intrinsically disordered protein regions and disorder-related functional sites. The program is particularly useful for detecting disordered regions and disorder-associated binding motifs [33]. Together, these complementary approaches provide a robust framework for analyzing IDRs in MHC-I molecules.

**Figure 2:**
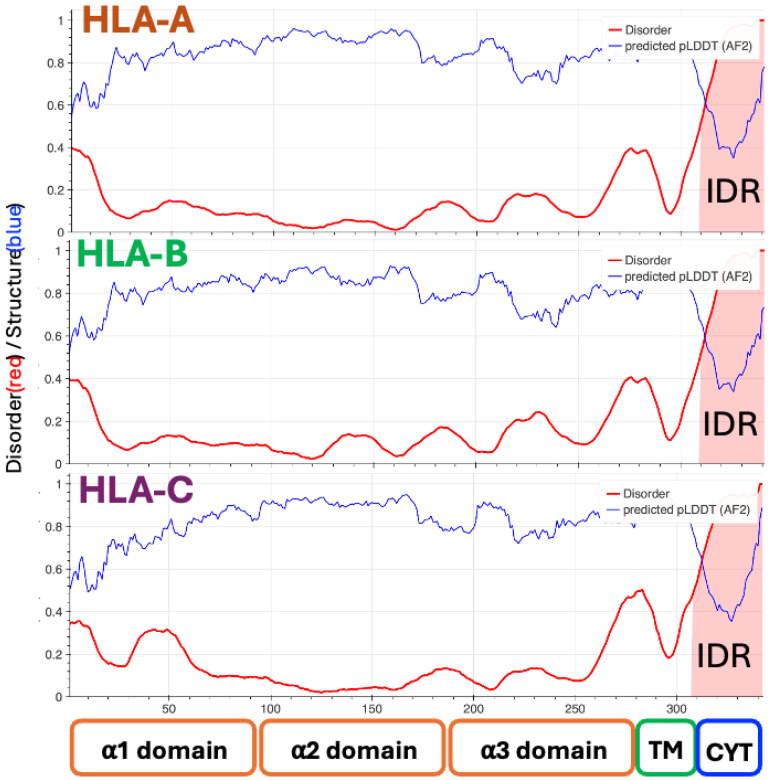
Intrinsic disorder analysis of consensus HLA-A, HLA-B, and HLA-C amino acid sequences using the MetaPredict algorithm, see ref. [25]. Red line indicates predicted intrinsic disorder. Regions mapped by this line indicate segments of the protein that lack a fixed, stable 3D structure and are highly dynamic [25]. Blue line indicates predicted Local Distance Difference Test (pLDDT) and AlphaFold 2. It is a per-residue confidence score that measures how confident the AI is in the predicted position and orientation of each specific amino acid. pLDDT ranges from 0 to 100. Both sets of values are plotted on a scale from (0 to 1). The α1, α2, α3, transmembrane (TM) and Cytoplasmic (CYT) domains are indicated. For disorder, regions scoring above 0.5 are considered intrinsically disordered, while those below 0.5 are considered structurally ordered. For structure, regions with values > 0.7–0.9 indicate that the region is intrinsically disordered or highly flexible (does not have a fixed 3D structure on its own). Regions with values < 0.5 suggest areas where the amino acid sequence tends to form rigid, stable, or folded protein structures (like α-helices or β-sheets). IDR: intrinsically disordered region.

**Figure 3:**
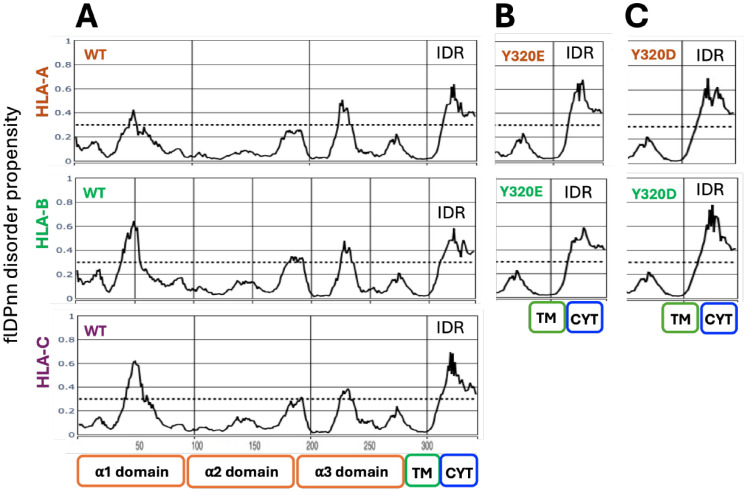
Intrinsic disorder analysis of consensus HLA-A, HLA-B, and HLA-C amino acid sequences using the flDPnn algorithm see ref. [26]. (**A**) The graphs show the disorder profiles of wild-type HLA-A, HLA-B, and HLA-C molecules, with indication of the α1, α2, α3, transmembrane (TM) and Cytoplasmic (CYT) domains. In addition to the cytoplasmic IDR identified by MetaPredict (see Figure 2), flDPnn predicted regions of increased disorder propensity within the extracellular region, namely in the α1 and α3 domains, in an allele-specific manner. (**B**) The graphs show the effect of the phosphomimetic substitution Y320E on the predicted disorder profile for HLA-A and HLA-B, showing only the TM and CYT domains. (**C**) The graphs show the effect of the phosphomimetic substitution Y320D on the predicted disorder profile for HLA-A and HLA-B, showing only in the TM and CYT domains. Residues scoring above 0.3 are considered intrinsically disordered, while those below 0.3 are considered structurally ordered.

Consensus amino acid FASTA sequences of the three classical mature human molecules, HLA-A, HLA-B, and HLA-C, were retrieved from the UniProt database (IDs: P04439, P01889, and P10321) and analyzed using the online MetaPredict (https://MetaPredict.net, 1 May 2026) and flDPnn (https://biomine.cs.vcu.edu/servers/flDPnn, 1 May 2026) servers. In MetaPredict, values above 0.5 indicate structurally disordered regions, whereas in flDPnn the threshold is 0.3. Intrinsic disorder analysis of HLA class I heavy chains revealed differences among the prediction tools. MetaPredict identified one major IDR within the C-terminal cytoplasmic domains of all three molecules studied (Figure 2). flDPnn corroborated the results obtained with MetaPredict and additionally identified a second prominent IDR within the α1 domain and a third, less prominent IDR, within the α3 domain (Figure 3A). The prediction that the α1 domain contains an IDR is not unexpected given the known flexibility of this domain [2,5]. The enrichment of disorder within the C-terminal cytoplasmic region of HLA-I molecules likely reflects functional requirements rather than structural instability. Indeed, IDRs are known to facilitate protein–protein interactions, post-translational modifications, and conformational adaptability [34,35]. Nevertheless, these analyses should be regarded as hypothesis-generating rather than definitive evidence of intrinsic disorder.

The disorder prediction results raised the important question of whether phosphorylation of the conserved Tyr320 residue affects the degree of disorder within the cytoplasmic tail. Previous studies have shown that phosphorylation of conserved tyrosine residues in several proteins induces conformational changes that may facilitate the binding of downstream signaling proteins [31,36,37]. To address this question, we used phosphomimetic substitutions to assess the effects of replacing Tyr320 with either glutamic acid (Y320E) or aspartic acid (Y320D) on the predicted IDRs using the flDPnn computational program. Glutamic acid and aspartic acid are commonly used as phosphomimetic substitutions because their negatively charged side chains partially mimic the electrostatic effects introduced by phosphorylation. These substitutions were employed to explore whether mimicking Tyr320 phosphorylation could alter the predicted disorder propensity of the HLA-A and HLA-B cytoplasmic domains. Although the non-phosphorylatable Y320F mutant used in previous studies [26,27] addresses a different mechanistic question (e.g., endocytosis) it was included in the present analysis. We selected flDPnn because, although MetaPredict is an excellent tool for identifying disordered regions, it is less suitable for assessing the effects of single amino acid substitutions. In contrast, flDPnn is sufficiently sensitive to detect how single-point mutations alter local disorder propensity [33], and can therefore be used to determine whether the phosphomimetic substitutions Y320E and Y320D increase or decrease local flexibility within the cytoplasmic tail. Although phosphomimetic substitutions have inherent limitations, they have recently been employed in functional studies of MHC-I molecules in antigen-presenting cells [26].

The Y320E substitution did not alter the disorder scores of the cytoplasmic domains of HLA-A and HLA-B when compared with their respective wild-type molecules (Figure 3B). In contrast, the Y320D phosphomimetic mutation increased the disorder score of the HLA-B, but not HLA-A, cytoplasmic domain by approximately 0.2 (in a scale of 0–1) when compared with wild-type HLA-B (Figure 3C). Analysis of the residues immediately upstream and downstream of position 320 containing the phosphomimetic residue aspartic acid (i.e., GGSDSQA) in HLA-B, revealed that Ser321 exhibited the largest increase in disorder propensity, suggesting that the Y320D substitution acts as a disorder-promoting mutation specifically in the context of the flexible serine residue at position 321 (see Figure 1A). In contrast, the residue immediately downstream of Tyr320 in HLA-A is Thr321 (see Figure 1A), a β-branched amino acid containing an additional methyl group, which may restrain conformational disorder. As a control, we also examined the non-phosphorylatable Y320F substitution, which, unlike the phosphomimetic Y320D substitution, did not alter the predicted disorder score of the cytoplasmic tail (i.e., disorder score changed by <0.02, essentially unchanged), supporting the specificity of the disorder-promoting effect of the phosphomimetic residue aspartic acid at position 320 (i.e., Y320D).

Collectively, the analyses presented in Figure 2 and Figure 3 suggest that the MHC-I cytoplasmic domain possesses features characteristic of intrinsically disordered regions and that phosphomimetic substitutions of the tyrosine at position 320 may alter its predicted disorder propensity in an allele-dependent manner. These predictions provide a useful framework for formulating mechanistic hypotheses and support the hypothesis that phosphorylation-dependent changes in the cytoplasmic tail could modulate MHC-I conformational dynamics and, consequently, intracellular trafficking, and signaling functions. Nevertheless, experimental approaches such as NMR, circular dichroism, phospho-specific analyses, and mutational studies will be required to validate the predicted disorder and establish its functional relevance.

## 5. The Central Nervous System as a Biological Context for Studying MHC-I Conformational Switching

Intrinsically disordered proteins and intrinsically disordered regions have been implicated in numerous human diseases, including cancer, cardiovascular disease, amyloidosis, diabetes, and neurodegenerative disorders [29,38]. Among neurodegenerative diseases, Alzheimer’s disease and Parkinson’s disease are particularly noteworthy because they are characterized by the accumulation of aggregation-prone proteins, including Aβ, α-synuclein, and Tau, which disrupt cellular function [38]. Unfortunately, no data are currently available regarding the involvement of cytoplasmic IDRs of classical MHC-I molecules in these pathological processes. Nevertheless, the central nervous system (CNS) provides an attractive context in which to evaluate the present model because reverse MHC-I signaling has already been implicated in the physiology of cells within the brain parenchyma. Here, the term MHC-I reverse signaling denotes the transmission of signals from extracellular MHC-I conformational states or ligand engagement to intracellular signaling pathways through the MHC-I cytoplasmic domain. In neuronal cells, reverse signaling modulates the activity of receptors that are important for brain homeostasis, including the insulin receptor, the fibroblast growth factor receptor 1, the glutamate receptor, and the Triggering Receptor Expressed on Myeloid cells 2 (TREM2) [39,40,41,42].

Reverse MHC-I signaling, also referred to as outside-in signaling, occurs in both immune and non-immune cell types and can influence important cellular processes, including activation, proliferation, programmed cell death, migration, and cytotoxicity [43,44]. Therefore, considering that neuronal MHC-I molecules contain conserved SLiMs within their cytoplasmic domains and that one of these motifs may influence the degree of intrinsic disorder following phosphorylation (see above), it can be proposed that changes in the expression of closed and open MHC-I conformers in non-immune cells, including neurons and glial cells, may influence reverse signaling. Importantly, the reverse MHC-I signaling may be further modulated by changes in the level of expression of MHC-I molecules. In that respect, a recent study, the Neurolipid Atlas Study, has shown that astrocytes and microglia derived from human iPS cells carrying the AD risk allele ApoE4 showed a significant downregulation of classical and non-classical HLA-I molecules. In marked contrast, reactive astrocytes showed the opposite phenotype, i.e., they upregulated the HLA class I machinery [45]. An increase in HLA-I expression may favor the accumulation of open conformers at the cell-surface and the release of soluble forms of HLA-I conformers akin to β2m [24,27], with potential consequences for neuroimmune communication [46,47]. Nevertheless, the functional relevance of classical MHC-I conformational switching and its direct relationship to neurodegenerative pathology remain largely unexplored.

At present, evidence directly linking MHC-I conformational switching and intrinsic disorder to neurodegenerative processes remains limited. Nevertheless, accumulating studies implicating MHC-I molecules in synaptic plasticity, neurogenesis, and neuroregeneration suggest that the physiological equilibria between closed and open MHC-I conformers, i.e., the conformational dynamics, may influence CNS homeostasis (ref. [48], and references therein). This possibility should therefore be regarded as a conceptual framework that generates experimentally testable hypotheses rather than an established mechanistic model. Future studies examining the distribution of MHC-I conformers, cytoplasmic tail phosphorylation, and reverse signaling in in vivo and in vitro experimental models of neurodegeneration may help determine whether these processes contribute to CNS dysfunction and aging-associated neurological disease.

Collectively, the current evidence supports the view that disruption of the physiological equilibrium between closed and open MHC-I conformers through altered expression, trafficking, or cytoplasmic-tail regulation may represent a mechanism contributing to age-associated immune and neurobiological decline [48]. However, direct causal relationships in both physiological and pathological settings remain to be established.

## 6. Concluding Remarks

Over recent decades, MHC-I molecules have evolved from being viewed solely as antigen-presenting structures to being recognized as dynamic transmembrane proteins with conformational and functional plasticity. The evidence discussed in this Perspective supports a unifying framework in which extracellular conformational states of MHC-I molecules are dynamically linked to cytoplasmic tail phosphorylation, intracellular trafficking, and reverse signaling, thereby positioning the MHC-I cytoplasmic domain as a potential molecular switch that coordinates these processes. Central to this model is the concept that transitions between closed and open conformers reflect regulated and reversible processes rather than passive structural instability. These processes are controlled, at least in part, by phosphorylation-dependent modifications of conserved residues embedded within SLiMs present in the cytoplasmic tail, particularly Tyr320.

In this context, the cytoplasmic tail functions as a regulatory hub that integrates extracellular structure with intracellular fate. Within this framework, open MHC-I conformers represent functionally distinct molecular states characterized by altered interaction capabilities, trafficking behavior, and signaling potential. These properties extend the role of MHC-I molecules beyond classical antigen presentation to broader functions in cellular communication and tissue-specific regulation, particularly in the immune system and the central nervous system.

Importantly, the model proposed here reconciles previously competing views by integrating plasma-membrane-based conformational equilibria with endocytic and recycling pathways, all governed by a shared regulatory architecture centered on cytoplasmic-tail signaling. Although emerging evidence suggests that dysregulation of this system may contribute to aging-associated immune and neurological alterations, definitive mechanistic links remain to be established. Several experimental approaches could be used to test the proposed molecular switch model. These include the generation of phosphomimetic and non-phosphorylatable mutants of Tyr320 and Ser335, the use of conformation-specific antibodies to monitor extracellular MHC-I conformers, phospho-specific analyses of the cytoplasmic tail, and structural and biophysical studies aimed at determining whether cytoplasmic tail modifications induce long-range conformational changes in MHC-I molecules. In addition, live-cell imaging and endocytic trafficking studies may help determine whether phosphorylation events and conformational transitions are causally linked or participate in reciprocal feedback mechanisms. These experimental approaches should help identify the molecular partners and signaling pathways, may help determine whether phosphorylation-dependent changes in the cytoplasmic tail influence β2m dissociation, peptide release, and the generation of open MHC-I conformers, and distinguish physiological contexts that determine how MHC-I conformational states are regulated and how they influence cellular and tissue function.

## Data Availability

The original contributions presented in this study are included in the article. Further inquiries can be directed to the corresponding authors.

## Abbreviations

flDPnn	function-linked Disorder Predictor and neuronal network
HLA-I	Human Leucocyte Antigens Class I
IDPs	Intrinsically Disordered Proteins
IDRs	Intrinsically Disordered Regions
MHC-I	Major Histocompatibility Molecules Class I
PTM	Post-Translational Modification
SLiMs	Short Linear Motifs
